# Diluting and Washing the Blood in Certain Forms of Poisoning

**Published:** 1903-06

**Authors:** W. W. Greer

**Affiliations:** Cameron, Texas


For Texas Medical Journal.

### Diluting and Washing the Blood in Certain Forms of Poisoning.

BY W. W. GREER, M. D., CAMERON, TEXAS.

The thoughts in this paper are not based on any experimentation whatsoever. Many of the poisons are effective through the medium of the circulation, poisoning every drop of blood in the system, and through this means impressing most profoundly the nervous system. Take for instance the poisons which slowly but surely kill when the lethal dose is taken, viz.: Morphine and chloral. Here is had an impression on the tissues of the blood, which is maintained for hours with coma, more or less profound. It occurs to me that if these agencies, at least in their effects, can, for such lengths of time, be suspended in the blood, it would afford an opportunity to come to the aid of nature and assist in relief by alternate blood-letting and transfusion, thereby diluting and minimizing their effects.

It is generally agreed that the normal saline solution is a good substitute for the blood. Why not extract the blood largely and replace it with this agent? This might be done by transfusing the saline solution in one arm while the blood was allowed to flow from the other, or perhaps the saline solution might be percolated into the cellular tissue, while the blood was being withdrawn from a small artery. There can be no fear from blood-letting under these conditions, since we may have at our easy command the means for raising the blood pressure and thus effectually counteracting the depression which naturally follows venesection. I have not seen a case of morphine poisoning for a long while, but I am resolved to try the above treatment should opportunity present itself.

If the treatment should shorten the long, weary hours usually required in watching a case of morphine poisoning, the advancement of this idea will need no apology.

---

*Read before the Brazos Valley Medical Association at its meeting at Cameron, Texas, May 12-13, 1903.

